# Contagious fear: Escape behavior increases with flock size in European gregarious birds

**DOI:** 10.1002/ece3.5193

**Published:** 2019-04-26

**Authors:** Federico Morelli, Yanina Benedetti, Mario Díaz, Tomas Grim, Juan Diego Ibáñez‐Álamo, Jukka Jokimäki, Marja‐Liisa Kaisanlahti‐Jokimäki, Kunter Tätte, Gábor Markó, Yiting Jiang, Piotr Tryjanowski, Anders Pape Møller

**Affiliations:** ^1^ Department of Applied Geoinformatics and Spatial Planning, Faculty of Environmental Sciences Czech University of Life Sciences Prague Prague Czech Republic; ^2^ Department of Biogeography and Global Change Museo Nacional de Ciencias Naturales (BGC‐MNCN‐CSIC) Madrid Spain; ^3^ Department of Zoology and Laboratory of Ornithology Palacky University Olomouc Czech Republic; ^4^ Behavioral and Physiological Ecology Group, Centre for Ecological and Evolutionary Studies University of Groningen Groningen The Netherlands; ^5^ Nature Inventory and EIA‐services, Arctic Centre University of Lapland Rovaniemi Finland; ^6^ Department of Zoology, Institute of Ecology & Earth Sciences University of Tartu Tartu Estonia; ^7^ Ecology Research Group, Hungarian Academy of Sciences, Hungarian Natural History Museum Eötvös Loránd University Budapest Hungary; ^8^ Behavioural Ecology Group, Department of Systematics, Zoology and Ecology Eötvös Loránd University Budapest Hungary; ^9^ Department of Plant Pathology Szent István University Budapest Hungary; ^10^ Ecologie Systématique Evolution, Université Paris‐Sud, CNRS, AgroParisTech Université Saclay Orsay France; ^11^ Institute of Zoology Poznań University of Life Sciences Poznan Poland

**Keywords:** birds, dilution effect, fear response, FID, gregariousness, human disturbance, social interactions, vigilance

## Abstract

Flight initiation distance (FID), the distance at which individuals take flight when approached by a potential (human) predator, is a tool for understanding predator–prey interactions. Among the factors affecting FID, tests of effects of group size (i.e., number of potential prey) on FID have yielded contrasting results. Group size or flock size could either affect FID negatively (i.e., the dilution effect caused by the presence of many individuals) or positively (i.e., increased vigilance due to more eyes scanning for predators). These effects may be associated with gregarious species, because such species should be better adapted to exploiting information from other individuals in the group than nongregarious species. Sociality may explain why earlier findings on group size versus FID have yielded different conclusions. Here, we analyzed how flock size affected bird FID in eight European countries. A phylogenetic generalized least square regression model was used to investigate changes in escape behavior of bird species in relation to number of individuals in the flock, starting distance, diet, latitude, and type of habitat. Flock size of different bird species influenced how species responded to perceived threats. We found that gregarious birds reacted to a potential predator earlier (longer FID) when aggregated in large flocks. These results support a higher vigilance arising from many eyes scanning in birds, suggesting that sociality may be a key factor in the evolution of antipredator behavior both in urban and rural areas. Finally, future studies comparing FID must pay explicit attention to the number of individuals in flocks of gregarious species.

## 1 INTRODUCTION

Flocking is an important behavior in birds, constituting also antipredator behavior by prey. In general, animals in larger groups will detect predators earlier (many/multiple eyes/ears or early‐warning hypothesis) (Lazarus, 1979). Individual group members have a lower probability of being caught by a predator (“dilution hypothesis”) in larger groups (Lima, 1995; Lima & Dill, 1990; Ydenberg & Dill, 1986). Other advantages of being a member of a large group are that individuals spend more time feeding and less time vigilant as group size increases (Lima & Dill, 1990) and that large groups could quickly respond to new situations (Liker & Bókony, 2009). Foraging prey that have noticed a predator should make a decision, either stay or escape, thereby balancing possible benefits (e.g., decreased capture probability) and costs (e.g., abandoning a food patch, reduced time spent foraging, and increased energy use for locomotion) (Frid & Dill, 2002). Many studies have indicated that prey are more vigilant when predation risk is high (Caro, 2005; Frid & Dill, 2002).

Flight initiation distance (FID) is defined as the distance at which animals take flight from approaching threats (Blumstein, 2013; Hediger, 1934). This behavioral trait has been used as a surrogate for antipredator or fear behavior in many ecological studies (Blumstein, 2006; Glover, Weston, Maguire, Miller, & Christie, 2011; Legagneux & Ducatez, 2013; Møller, 2008a; Møller, Grim, Ibáñez‐Álamo, Markó, & Tryjanowski, 2013; Weston, Mcleod, Blumstein, & Guay, 2012). Briefly, this measure indicates when individuals take more risk (i.e., delayed escape) or take less risk (i.e., escape earlier) (Sol et al., 2018). Many studies of escape behavior in birds focused on the influence of external factors affecting behavioral responses: habitat quality (Burger, Gochfeld, Jenkins, & Lesser, 2010), the direction of approach by predators (Møller & Tryjanowski, 2014), intruder starting distance (Blumstein, 2013; Glover et al., 2011), number or density of intruders (Geist, Liao, Libby, & Blumstein, 2001), population density (Mikula, 2014), urbanization (Samia et al., 2017), road speed limits (Legagneux & Ducatez, 2013), insular distribution (Cooper, Pyron, & Garland, 2014), predator–prey interactions (Møller, 2008b), spatial gradients of predator abundance (Díaz et al., 2013), or daytime and season when FID was measured (Burger & Gochfeld, 1991; Piratelli, Favoretto, & de Almeida Maximiano, 2015). Blumstein (2006) has made links between escape behavior and life history and natural history traits (e.g., diet) in birds. Furthermore, earlier studies assumed that animals will respond to human approach in a similar way as they do when responding to predation (Bötsch, Gugelmann, Tablado, & Jenni, 2018; Frid & Dill, 2002; Møller & Tryjanowski, 2014; Morelli et al., 2018; Weston et al., 2012).

According to Ydenberg and Dill (1986), FID increases with the risk of capture and the increasing cost of flight. Large species of birds have long FIDs, because larger species need more time to get airborne and hence avoid capture (Fernández‐Juricic et al., 2006; Hemmingsen, 1951; Møller, 2008c; Weston et al., 2012). We know that birds from rural areas tend to escape earlier than birds from urban areas, being less tolerant of humans, probably because urban birds live under lower predation risk than their rural counterparts (Møller, 2015; Samia et al., 2017), because urban birds have become adapted or habituated to the presence of humans (Carrete & Tella, 2013; Holtmann, Santos, Lara, & Nakagawa, 2017), or because local selection for bolder individuals has occurred (van Dongen, Robinson, Weston, Mulder, & Guay, 2015). Additionally, we know that behavioral responses of animals to human approach such as FID can be useful for conservation purposes, namely management of disturbance, especially in human‐dominated environments (Guay, Dongen, Robinson, Blumstein, & Weston, 2016; Weston et al., 2012). However, we know very little about the intraspecific factors that can be involved in variation in FID. Group size has been suggested to be another important component that influences escape decisions by prey (Burger & Gochfeld, 1991; Fernández‐Juricic, Jimenez, & Lucas, 2002; Glover et al., 2011; Samia et al., 2017; Yasué, 2005). However, the relationships between group size and FID have been diverse (Deboelpaep, Keleman, Vanschoenwinkel, & Koedam, 2018; Lima & Dill, 1990; Ydenberg & Dill, 1986). According to the early‐warning hypothesis, a larger flock will flee earlier, that is, having a longer FID, because it will detect a predator earlier despite per capita decreases in vigilance rates. However, according to the dilution hypotheses, the cost of remaining may be smaller in larger flocks, that is, FID will be reduced. It is also possible that if foraging efficiency is superior in larger than in smaller groups, then any response of the predator may be delayed, causing a shorter FID.

Predation has been shown to be an important selective force affecting patterns of sociality, such as grouping (Lima & Dill, 1990). Vigilance in response to predators as a social phenomenon has been studied intensively as a component of antipredator behavior (Caro, 2005). In general, members in large groups spend less time vigilant (Caro, 2005; Lima & Dill, 1990). However, a large amount of variation in the relationships between group size and vigilance remains unexplained, for example, due to the spatial position of group members in the flock, dominance status, sex, and probably other factors (Beauchamp, 2008; Ydenberg & Dill, 1986). It is also possible that vigilance could firstly decline and thereafter increase when group size increases (Wang, Li, Beauchamp, & Jiang, 2011). Møller (2015) reviewed the literature on FID in birds and showed that sociality is an important factor influencing FID. There is less information available for other classes of animals (Cooper & Blumstein, 2015). Under classical ecological models of predation risk, which predict a decrease in individual risk when group size increases (Alexander, 1974; Pulliam, 1973), we would expect a reduction in FID among individuals in large groups through the dilution effect (Fernández‐Juricic et al., 2002; Pulliam, 1973; Roberts, 1996). However, the opposite response could also be expected (increasing FID with increasing flock size) because fear responses may be socially transmitted (Griffin, 2004), as is early detection of predators by large groups (Hingee & Magrath, 2009; Stankowich & Blumstein, 2005). Awareness or nervous reactions can be positively related to flock size because of higher vigilance (effect of many eyes scanning for predators) (Pulliam, 1973). Thus, under threat, individuals in large flocks should react more rapidly than solitary individuals or individuals in small groups, as a consequence of cascade effects or contagious alertness.

Although the literature on fear responses and sociality is limited, cooperative breeders are known to be more alarmed than species with other breeding systems (Blumstein, 2006), which is consistent with the second hypothesis. The study by Laursen, Kahlert, and Frikke (2005) showed that, in different species of waterbirds, FID increased with flock size. These findings are inconsistent with dilution effects, because if each individual in a flock experienced a smaller risk, we should expect a shorter FID in larger flocks. In contrast, the results are consistent with effects of many eyes scanning for the presence of a predator, although differences in phenotypic composition of differently sized flocks may be an alternative explanation for these findings. Finally, Tätte, Møller, and Mänd (2018) showed that flock size increased FID, but not the distance fled.

Gregariousness is common in nature and can be defined as the tendency to live in flocks (Miller, 1922). A flock is a term used to define any aggregation of homogeneous individuals, that live, travel, or feed together, regardless of size or density (Emlen, 1952). As pointed out by Miller (1922), probably the most obvious advantage of gregarious behavior in birds is that it affords a multiplicity of eyes, increasing the probability of sighting a potential predator or prey. Thus, any defensive measures can be taken early, increasing the probability of successful escape when encountering a risk (Miller, 1922).

We hypothesized that FID in response to human approach would be longer in individuals aggregated in large flocks. The ecological rationale for this expectation is that vigilance and FID increase with group size because of the many eyes effect, and vigilance can be transmitted more easily (or quickly) when there are many individuals in a flock. As a consequence, the aim of this study was to test whether FID in birds increases with the number of conspecifics (flock size), focusing on differences between environments, latitudes, and species’ traits such as diet. We explored differences in FID between environments because previous studies suggested significant differences between urban and rural birds (Díaz et al., 2013; Piratelli et al., 2015; Samia et al., 2017). Additionally, we focused on potential differences associated with diet in an effort to test whether foraging strategies can affect the escape behavior of species. Flock size was measured as the number of individuals of the same species aggregated in a group. We focused on gregarious bird species because such species tend to stay in groups. In this study, we used body mass‐corrected FID throughout, because large‐bodied species require more effort to get airborne.

## 2 METHODS

### 2.1 Study area and flight initiation distance

**Figure 1:**
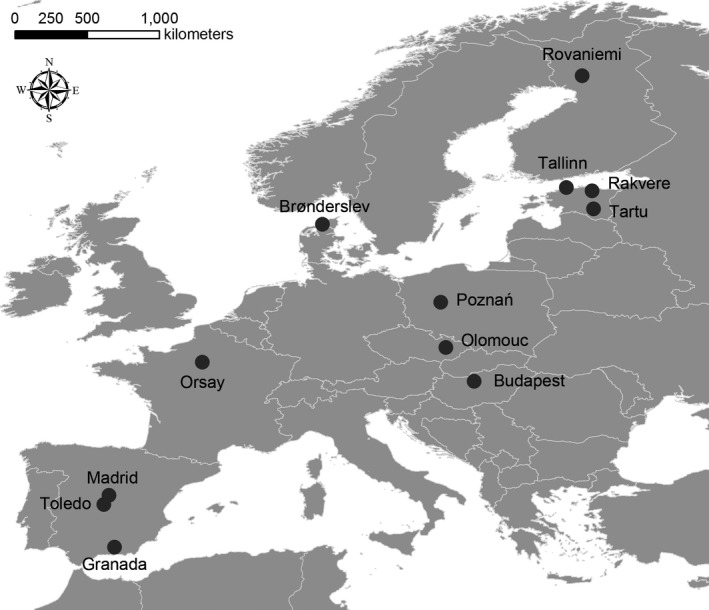
Location of 12 study sites across eight European countries, where data on flight initiation distance (FID) of gregarious birds were collected. Each site contained one urban and one nearby rural location

Data were collected during the breeding period in each study area (April–September 2015) using a standard protocol (Blumstein, 2006; Samia et al., 2017) in urban and adjacent rural areas of eight cities in eight European countries: Czech Republic, Denmark, Estonia, Finland, France, Hungary, Poland, and Spain (Figure 1; Table S1). Because the wide latitudinal gradient in our study, FID data were collected in all localities during a comparable period using a narrow temporal phenological window according to latitude, in order to control any effect of seasonality (Weston, Ju, Guay, & Naismith, 2018). For the same reason, we focused our study almost exclusively on adult individuals during the peak of the breeding season, in each country.

We used a study design collecting data in urban and rural sites (habitat type) in each study location, because a large amount of literature highlights the main differences between urban and rural environments, in terms of responses of birds to risk of predation (Møller, 2012; Møller et al., 2013; Samia et al., 2017; Sol et al., 2018). The distance between each pair of urban and rural site was always shorter than 20 km (with a minimum of 3.5 km). The sites classified as “urban” were characterized by areas with multistory buildings or by areas with single‐family houses (suburban areas). The sites classified as “rural” were dominated by open farmland with scattered houses (Samia et al., 2017). For the classification of urban and rural habitats, we followed the definitions provided in Marzluff, Bowman, and Donnelly (2001). Urban habitats were defined as areas with at least 50% built‐up area, building density >10 buildings/ha, and a residential human density >10 humans/ha. Rural habitats were defined as the areas with 5%–20% built‐up areas, a building density <2.5 buildings/ha, and residential human density between 1 and 10 humans/ha (Marzluff et al., 2001).

Observers used binoculars to identify birds that were foraging or engaged in “relaxed behavior” (i.e., roosting or preening). Flight initiation distance observation is considered reliable even when collected by different observers (Guay, McLeod, et al., 2013b). Highly vigilant or obviously alarmed individuals were not approached. Furthermore, data from breeding sites (e.g., from gull colonies) or anthropogenic feeding sites (e.g., rubbish dumps) were not collected, in order to reduce disturbance (breeding sites) or avoid an excessive effect of artificial food source on the behavior of birds. Each individual bird was approached in a straight line by the observer walking at a constant speed (0.5 m/s). Starting distance was measured as the distance at which an observer started the approach to the bird, in meters (Blumstein, 2013). Flight initiation distance was measured as the distance between the observer and the point where the individual bird began to flee (see more details in Samia et al., 2017). Only bird species detected on the ground were considered. Sol et al. (2018) showed that species with more than 10 recorded cases per study site provided reliable estimates of FID. Here, we only selected bird species with more than 10 observations of FID. To avoid collecting the same flock several times, we visited every site just once.

Flock size was defined as the number of aggregated individuals of the same species, implying that individuals in flocks are closer to each other than individuals that are not in flocks. We collected data on FID for single individuals or well‐recognizable flocks, at a distance from other individuals or flocks longer than 10 m. Only single‐species flocks were targeted in this study, because mixed flocks could be problematic if the species present differ in their tolerance to humans. When birds were in a flock, we always selected the closest individual to the observer, because that individual generally would have the shortest FID.

### 2.2 Ecological variables: gregariousness, trophic guild, and body mass

In this study, we focused only on “gregarious” species because, by definition, such species can be found in aggregated groups. The gregariousness was classified using information from the Handbook of the Birds of the Western Palearctic (Cramp & Perrins, 1994) (Table S2). Birds were classified as “gregarious" when species have shown gregarious activities either during breeding or nonbreeding, following the classification made in the same book (Cramp & Perrins, 1994). There was a positive correlation between gregariousness during these two periods (Kendall rank order correlation *τ* = 0.40, *p* = 0.020), implying that species that were gregarious during breeding also tend to be gregarious during the nonbreeding season. We decided to group breeding and nonbreeding gregarious species, because we assumed that social cognition, that is, the capacity to communicate with other individuals belonging to the same species, is a species‐specific trait that may be manifest all the time (Yu et al., 2016, 2017)**.**

For each gregarious species recorded in this study, we included the following information: trophic guild or diet (main type of food consumed, following the bird traits of feeding ecology provided in Pearman et al. (2014)). All species were classified into five main categories as granivorous, granivorous–insectivorous, insectivorous, carnivorous, and carrion‐eater (Table S2). Body mass for each species was obtained from the same source (Pearman et al., 2014), and this variable was log‐transformed to fit normality.

### 2.3 Statistical analyses

The average values of FID and flock size between birds classified on the basis of their species‐specific gregariousness were compared using the standard nonparametric Wilcoxon test (Triola, 2012).

To test the presence of a phylogenetic signal (Blomberg & Garland, 2003) in FID data for gregarious bird species, we used Blomberg's *K* statistic (Blomberg, Garland, & Ives, 2003). When *K* approaches 1, trait evolution follows a mode of evolution that is consistent with Brownian motion, and if *K* > 1, close relatives are more similar than expected under Brownian motion and indicate a strong phylogenetic signal, while *K* values closer to zero correspond to a random or convergent pattern of evolution, and that closely related species are less similar than expected (Blomberg et al., 2003). Blomberg's *K* statistic was estimated using the R package “phylosignal” (Keck, Rimet, Bouchez, & Franc, 2016). To control for the phylogenetic relationship among species, we used phylogenetic generalized least square regression models to analyze the changes in FID behavior of bird species in relation to flock size and characteristics of species. Models were fitted using the package “ape” (Paradis, Claude, & Strimmer, 2004), “nlme” (Pinheiro, Bates, DebRoy, & Sarkar, 2017), and the function “gls” with correlation equals consensus tree. We extracted the phylogenetic relationship for all 23 species from the phylogeny available online (Jetz, Thomas, Joy, Hartmann, & Mooers, 2012; Jetz et al., 2014) and obtained consensus phylogenies from 100 random trees with Mesquite (Maddison & Maddison, 2018). Different populations of the same species from different countries and habitats were defined with a relatively different branch length of 1E−18 just to fit the models. We weighted models by sample size (see more details in Garamszegi (2014)). In order to reduce any effect associated with a strong correlation between FID and body mass (Møller, Samia, Weston, Guay, & Blumstein, 2016), we first did a log–log‐linear regression for FID and body mass and use the residuals of this model to represent relative FID. This allowed us to focus on the main effects of selected predictors. The full model considered relative FID as response variable, while flock size, starting distance, habitat type (urban or rural), latitude, and diet were introduced as predictors.

All statistical tests were performed with R software version 3.2.4 (R Development Core & Team, 2017).

## 3 RESULTS

From a total of 5,783 observations from eight different European countries (Figure 1; Table S1), all observations of FID for the 23 gregarious birds were collected with sample size large than 10 observations per species (Table S2).

**Figure 2:**
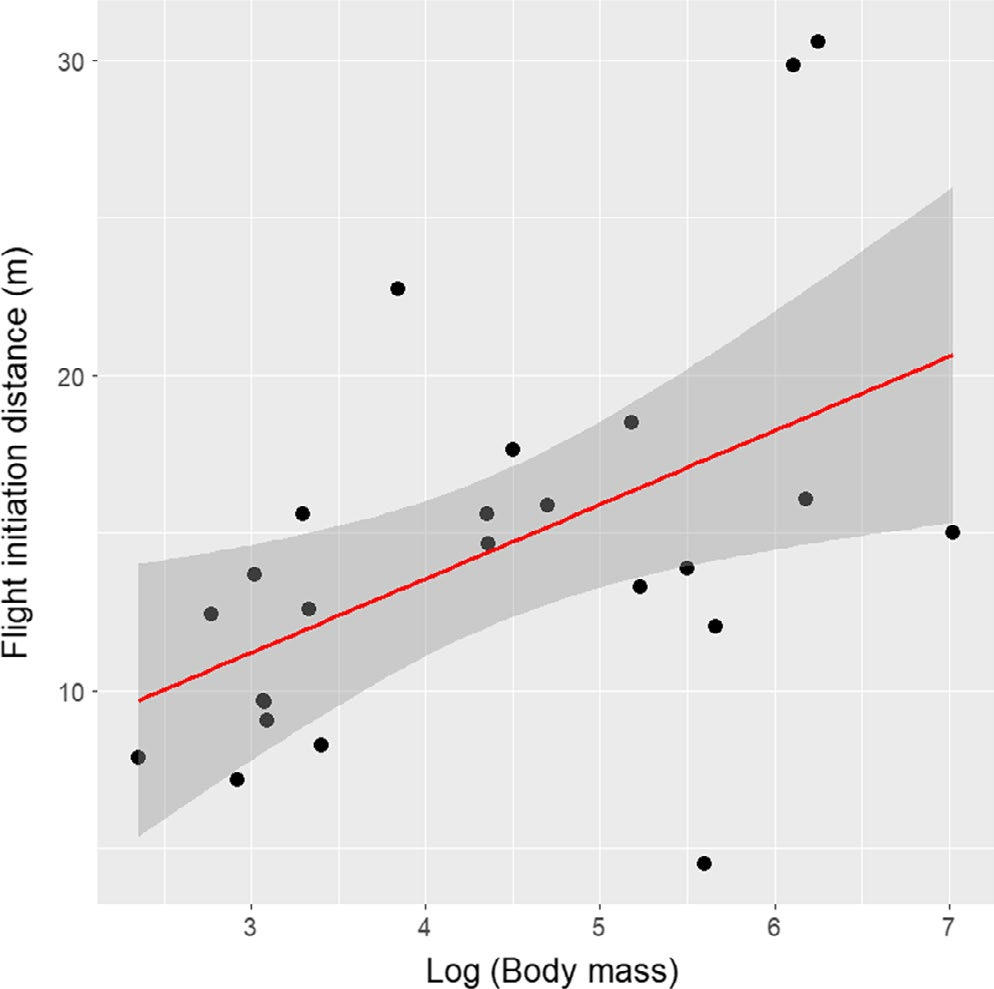
Linear regression lines between mean flight initiation distance (FID, m) and log‐transformed body mass (g) in all gregarious bird species recorded in this study in eight European countries. Envelopes around lines are 95% confidence intervals

In the initial exploration of data, FID was positively correlated with body mass (Figure 2). The FID for gregarious bird species ranged from a minimum of close to 0 m to a maximum of 152 m, with a mean value = 15.2 with *SD* = 13 m in rural habitat and 8.7 + *SD* = 7 m in urban habitats. A preliminary graphical exploration showed that FID was shorter in urban than in rural habitats for the majority of gregarious birds that were the focus of this study, with the only exceptions being *Corvus monedula* and *Parus caeruleus* (Figure S1).

**Figure 3:**
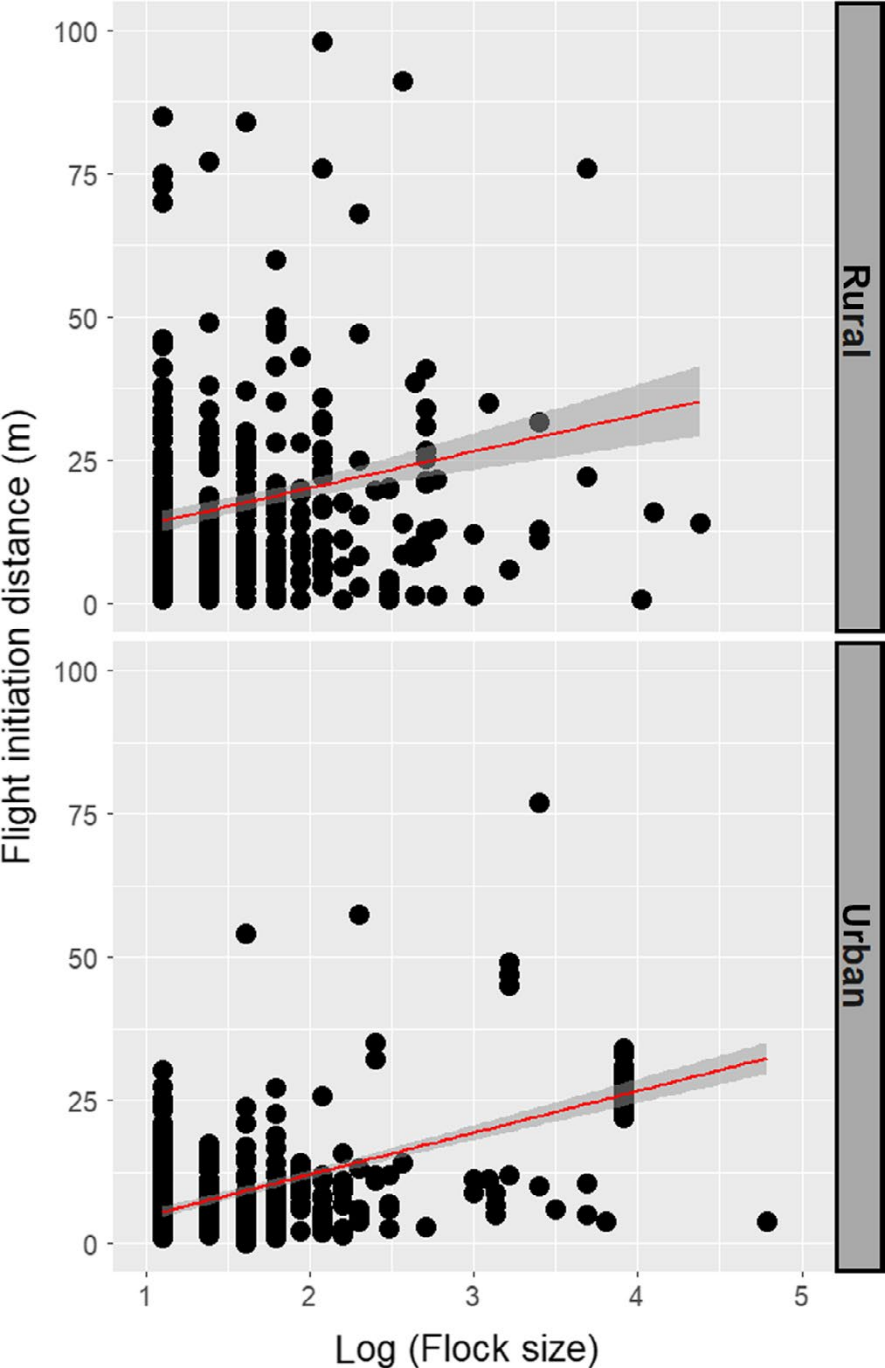
Linear regression lines between flight initiation distance (FID, m) and log‐transformed flock size in gregarious bird species from rural and urban environments in eight European countries. Envelopes around lines are 95% confidence intervals

The FID for gregarious bird species showed a strong phylogenetic signal with a *K* statistic approaching 1 and with *p* < 0.01, suggesting a model similar to Brownian motion. The result of a phylogenetic generalized linear regression model (PGLS) showed that relative FID of individuals from rural and urban habitats was positively associated with flock size (Figure 3) and starting distance, while relative FID was shorter in urban habitats and for granivorous–insectivorous and insectivorous species (Table 1). Latitude and granivorous diet were both unrelated to the values of relative FID (Table 1).

**Table 1:** Results of phylogenetic generalized linear regression model (PGLS), accounting for variation in relative flight initiation distance (FID) in relation to flock size, starting distance, habitat (urban or rural), latitude, and diet in gregarious bird species

Variables	Estimate	*SE*	*t*	*p*
(Intercept)	0.109	0.047	2.335	0.019
**Flock**	**0.007**	**0.001**	**6.220**	**<0.0001**
**Starting distance**	**0.005**	**0.001**	**10.163**	**<0.0001**
Latitude	−0.001	0.001	−1.218	0.223
**Habitat (urban)**	**−0.175**	**0.012**	**−14.084**	**<0.0001**
Diet (granivorous)	−0.064	0.038	−1.652	0.097
**Diet (granivorous**–**insectivorous)**	**−0.119**	**0.031**	**−3.877**	**0.0001**
**Diet (insectivorous)**	**−0.149**	**0.037**	**−3.992**	**0.0001**

Models were based on data from eight European countries, weighted by sample size for species of birds. Significant variables are highlighted in bold. Model statistics: residual standard error: 0.584, degrees of freedom: 1,146 total; 1,137 residual, *R* ^2^ = 0.202. *SE*: standard error.

## 4 DISCUSSION

The main finding of this study of FID in birds was that FID increased with flock size in European gregarious bird species, in rural and urban habitat. Gregarious species may be more susceptible to human disturbance than nongregarious species, both at the individual and probably at the population levels (Weston et al., 2012). Our statistical model also confirmed the positive association between FID and starting distance of observer, already shown in other studies (Blumstein, 2013). In addition, relative FID tended to decrease in urban habitats when compared with rural areas, confirming previous studies (Díaz et al., 2013; Møller et al., 2016; Samia et al., 2017; Weston et al., 2012). In this study on European gregarious birds, diet was significantly associated with relative FID, with insectivorous and granivorous–insectivorous species having the shortest relative FID (Figure S2). We believe that this association between foraging strategy or diet and escape behavior of birds deserves further study, as suggested also in a previous study (Blumstein, 2006).

We tried to test indirectly the two hypotheses presented in the Introduction: the dilution effect (Stankowich & Blumstein, 2005) and the many eyes effect (Hingee & Magrath, 2009; Stankowich & Blumstein, 2005). According to these hypotheses, larger flocks have more individuals scanning for predators, but larger flocks also result in greater dilution effects because the risk of mortality is smaller for each individual in a larger flock. Here, mainly using Passeriformes, we have shown a positive relationship between FID and flock size, which supports the many eyes effect hypothesis. A similar general pattern was previously reported by Laursen et al. (2005) for waterbirds and by Glover et al. (2011) for red‐necked stint *Calidris ruficollis*, while other studies suggested no influence of flock size on FID for the particular case of black swans *Cygnus atratus* (Guay, Lorenz, Robinson, Symonds, & Weston, 2013) or for other bird species (Fernández‐Juricic et al., 2006; Guay, McLeod, et al., 2013b) or negative association between flock size and FID for waders (Charadriiformes) (Mikula et al., 2018). The present study of mainly passerine birds compares well with that by Laursen et al. (2005) for waterbirds comprised of geese, ducks, waders, and gulls. In fact, both studies have very similar effect sizes despite the taxa being completely different, but the association with urbanization was focused only in our study. However, hunting activities affected FID in the study by Laursen et al. (2005), but not in our study in which only three of 23 focal species were hunted: mallard *Anas platyrhynchos*, wood pigeon *Columba palumbus,* and rook *Corvus frugilegus*. Indeed Laursen et al. (2005) showed an association between flock size and FID for nine waterbird species in fall, when hunting is common, but only for two species in spring when hunting ceased.

Vigilance in large groups can provide increased capacity to detect predators, thereby allowing individuals to spend additional time on foraging activities (Olson, Haley, Dyer, & Adami, 2015). Longer FID in larger flocks of a given species implies that individuals on average run higher risk in small flocks. This could either be due to such larger flocks being composed of individuals of lower phenotypic quality, or that individuals in small flocks with short FID run higher risks of mortality. Indeed, Møller (2014) has shown that barn swallow *Hirundo rustica* individuals with short FID are more likely to be caught by sparrowhawks *Accipiter nisus*. In addition, bird species with shorter FID are more vulnerable to predation by raptors (Møller, Nielsen, & Garamszegi, 2008) and cats *Felis catus* (Møller, Berthold, & Fiedler, 2010), but also to be killed by cars (Møller, Erritzøe, & Erritzøe, 2011). An alternative interpretation when measuring FID in flocks of many individuals could be that the first individual responder may set off a social escape response. In that case, FID would not reflect average tolerance of the group to predators, but rather the least tolerant individual in the flock (e.g., large flocks may flush earlier because there is a probability that they contain especially sensitive individuals). However, our study does not allow discrimination between these different hypotheses.

We assumed that gregarious species would encounter conspecifics more often than nongregarious species (Emlen, 1952). For this reason, we hypothesized that individuals of gregarious species are better adapted at extracting information from other individuals than nongregarious species, and then, their escape behavior could be affected by the number of surrounding individuals. The increased skill to exploit information from other individuals and signal effectively should improve the efficiency of the group at detection and defense against predators (Krebs, MacRoberts, & Cullen, 1972; Treisman, 1975). Thus, individuals belonging to gregarious species would experience a trade‐off between foraging (or resting) under the safety of the presence of many conspecifics and hence long FIDs, or such individuals may have short FIDs in the presence of few conspecifics (Laursen et al., 2005). Accordingly, our results highlighted that relative FID was positively associated with flock size in European gregarious birds.

We explicitly recorded FID observations from urban and rural habitats. While previous studies have shown consistently longer FIDs in rural than in urban habitats (Samia et al., 2017), we are only aware of a single other study investigating the independent effects of rural versus urban habitats and flock size on FID (Tätte et al., 2018). The latter study also found a similar effect of flock size on FID in rural and urban habitats. Our results suggested also that FID in urban areas tends to be shorter than in rural areas. However, the positive association between flock size and FID found for gregarious species was similar across the two types of habitats.

Our findings suggest that future studies on escape behavior of birds should explicitly consider flock size, at least in gregarious bird species. The influence of the many eyes effect in the presence of numerous conspecifics can significantly alter the escape behavior of social birds. We hypothesize that experimental change in sociality will affect FID. Indeed, Laursen, Møller, and Holm (2016) have shown that flock size changes adaptively in response to intense hunting. We predicted that such changes in immediate risk will be accompanied by similarly directed changes in FID. In conclusion, relative FID increased with flock size in gregarious species, independently of the rural versus urban areas. Our results support the role of sociality for risk‐taking behavior and hence for social organization. These conclusions have broad biological implications, especially considering the role of sociality (gregariousness) as possible factor facilitating colonization of urban environments and adaptation to such human‐impacted environments.

## ETHICS STATEMENTS

In this study, we did not capture or band birds, but only estimated of flight initiation distance (FID) based on observations of long‐distance behavior. The jurisdictions and institutions involved in this study required neither ethics clearance nor research permits for the noninvasive methodology employed.

## CONFLICT OF INTEREST

The author(s) declare not to have any conflict of interest or competing interests.

## AUTHOR CONTRIBUTIONS

F.M., Y.B., and A.P.M. planned the research. A.P.M., M.D., T.G., J.D.I.A., J.J., M.L.K.J, K.T., G.M., and P.T. collected data and curated the dataset. F.M., Y.B., and Y.J performed the data analyses and prepared the figures. F.M., Y.B, and A.P.M. wrote the first draft of the paper. All the authors contributed to the writing, and read and approved the final version of the manuscript.

## Data Availability

After manuscript acceptance, data will be archived in an appropriate public repository or journal website. Data available from the Dryad Digital Repository: https://doi.org/10.5061/dryad.3q25r77.
