# Supplementary material for: Contagious fear: Escape behavior increases with flock size in European gregarious birds
Source: Ecol Evol. 2019 Apr 26;9(10):6096–104. doi: 10.1002/ece3.5193 (PMC6540657; doi:10.1002/ece3.5193)

**Electronic Supplementary Material**

**Contagious fear: Escape behaviour increases with flock size in European gregarious birds**

Federico Morelli, Yanina Benedetti, Mario Díaz, Tomas Grim, Juan Diego Ibáñez-Álamo, Jukka Jokimäki, Marja-Liisa Kaisanlahti-Jokimäki, Kunter Tätte, Gábor Markó, Yiting Jiang, Piotr Tryjanowski and Anders Pape Møller

Table S1. List of cities and number of FID observations collected per type of habitat in eight European countries.

| **City** | **Country** | **Rural** | **Urban** | **Total FID observations** |
| --- | --- | --- | --- | --- |
| Brønderslev | Denmark | 231 | 137 | 368 |
| Budapest | Hungary | 266 | 337 | 603 |
| Granada | Spain | 226 | 295 | 521 |
| Madrid | Spain | 486 | 160 | 646 |
| Olomouc | Czech Republic | 237 | 215 | 452 |
| Orsay | France | 226 | 311 | 537 |
| Poznan | Poland | 395 | 145 | 540 |
| Rakvere | Estonia | 32 | 14 | 46 |
| Rovaniemi | Finland | 280 | 438 | 718 |
| Tallinn | Estonia | 102 | 223 | 325 |
| Tartu | Estonia | 198 | 210 | 408 |
| Toledo | Spain | 448 | 170 | 618 |
| Total |  | 3127 | 2655 | 5782 |

Table S2. Species name, body mass (g), diet, gregarious behavior for breeding and non-breeding seasons and family of gregarious birds species that are the focus in this study. The species were classified as “gregarious” considering gregariousness during breeding and non-breeding seasons, using information from the Handbook of the birds of the Western Palearctic (Cramp and Perrins, 1994). Breeding and non-breeding gregariousness values: 0 = no, 1 = yes.

| **Species** | **Body mass (g)** | **Diet** | **Breeding gregariousness** | **Non-breeding gregariousness** | **Family** |
| --- | --- | --- | --- | --- | --- |
| *Anas platyrhynchos* | 1125.0 | granivorous-insectivorous | 0 | 1 | Anatidae |
| *Carduelis carduelis* | 16.0 | granivorous | 0 | 1 | Fringillidae |
| *Columba livia* | 270.0 | granivorous-insectivorous | 1 | 1 | Columbidae |
| *Columba palumbus* | 485.0 | granivorous | 0 | 1 | Columbidae |
| *Corvus corone* | 517.6 | carrion-eater | 0 | 1 | Corvidae |
| *Corvus frugilegus* | 450.0 | carrion-eater | 1 | 1 | Corvidae |
| *Corvus monedula* | 245.0 | carrion-eater | 1 | 1 | Corvidae |
| *Emberiza citrinella* | 27.0 | granivorous-insectivorous | 0 | 1 | Emberizidae |
| *Fringilla coelebs* | 21.5 | granivorous-insectivorous | 0 | 1 | Fringillidae |
| *Hirundo rustica* | 20.5 | insectivorous | 1 | 1 | Hirundinidae |
| *Larus ridibundus* | 287.5 | carrion-eater | 1 | 1 | Laridae |
| *Miliaria calandra* | 46.5 | granivorous-insectivorous | 0 | 1 | Emberizidae |
| *Motacilla alba* | 21.7 | insectivorous | 0 | 1 | Motacillidae |
| *Parus caeruleus* | 10.5 | insectivorous | 0 | 1 | Paridae |
| *Parus major* | 18.5 | insectivorous | 0 | 1 | Paridae |
| *Passer domesticus* | 30.0 | granivorous-insectivorous | 1 | 1 | Passeridae |
| *Passer montanus* | 22.0 | granivorous-insectivorous | 1 | 1 | Passeridae |
| *Pica pica* | 177.5 | carrion-eater | 0 | 1 | Corvidae |
| *Streptopelia decaocto* | 187.5 | granivorous | 0 | 1 | Columbidae |
| *Sturnus unicolor* | 90.0 | granivorous-insectivorous | 1 | 1 | Sturnidae |
| *Sturnus vulgaris* | 78.0 | granivorous-insectivorous | 1 | 1 | Sturnidae |
| *Turdus philomelos* | 77.5 | granivorous-insectivorous | 0 | 1 | Turdidae |
| *Turdus pilaris* | 110.0 | granivorous-insectivorous | 1 | 1 | Turdidae |

Figure S1. Flight initiation distance (FID) of gregarious bird species that are the focus of this study, in rural and urban habitat in eight different European countries. The box plots show medians, quartiles, 5- and 95-percentiles, jittered points (small grey dots) and extreme values (colored dots). Mean values are indicated with black rhombus. Jitter is a graphical tool which assigns a small random value to each observation to improve the graphical representation.


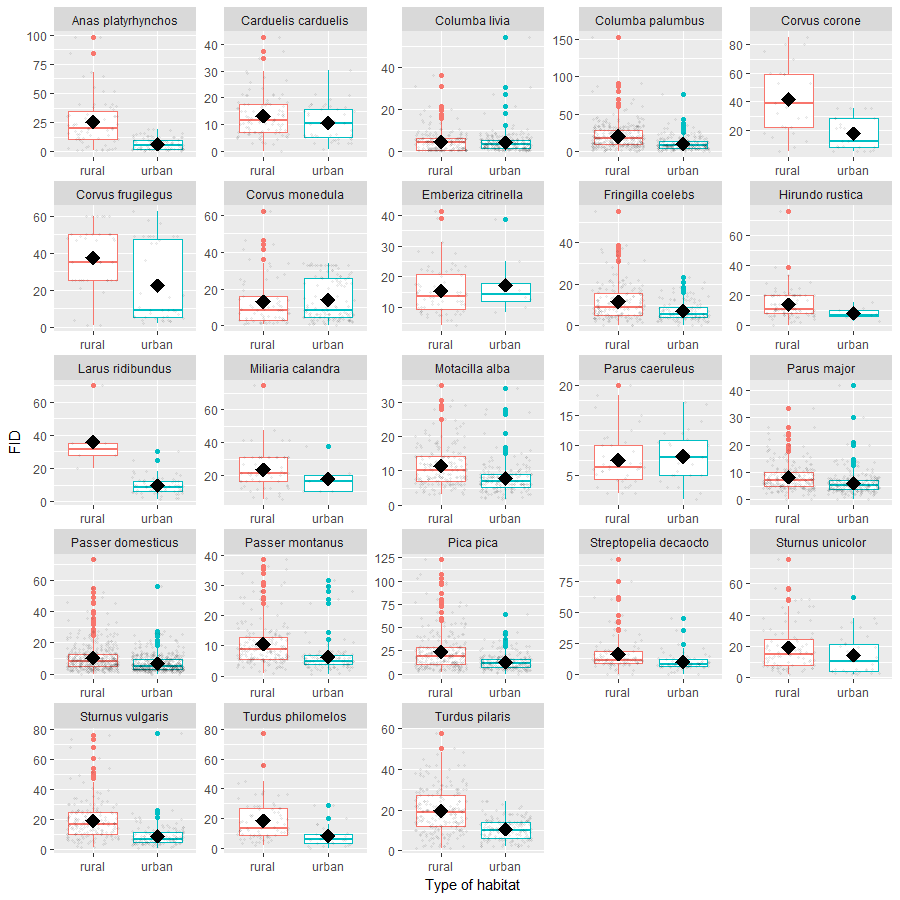


Figure S2. Flight initiation distance (FID) of gregarious bird species that are the focused of this study and type of diet. The box plots show medians, quartiles, 5- and 95-percentiles, jittered points (small grey dots) and extreme values (dots). Mean values are indicated with black rhombus. Jitter is a graphical tool which assigns a small random value to each observation to improve the graphical representation.


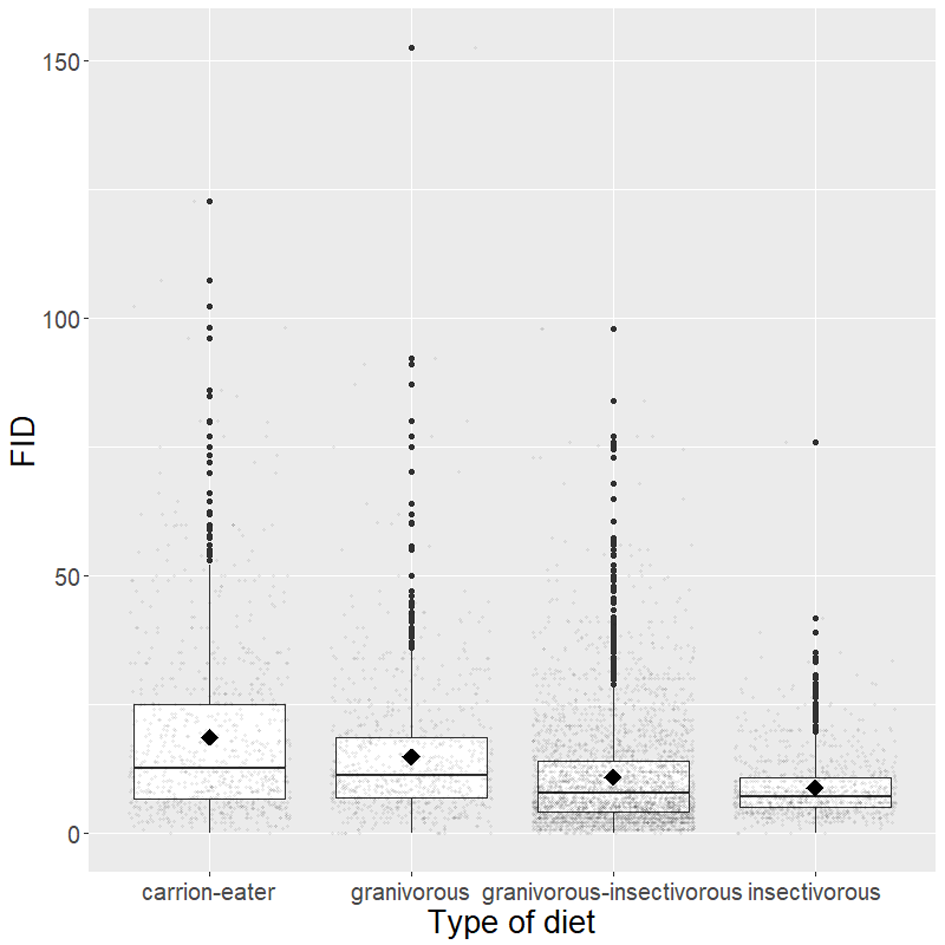

Supplement: Supplementary file 1 [file ECE3-9-6096-s001.docx]
